# Design and evaluation of sodium alginate-based hydrogel dressings containing Betula utilis extract for cutaneous wound healing

**DOI:** 10.3389/fbioe.2023.1042077

**Published:** 2023-01-26

**Authors:** Bushra Ishfaq, Ikram Ullah Khan, Syed Haroon Khalid, Sajid Asghar

**Affiliations:** Department of Pharmaceutics, Faculty of Pharmaceutical Sciences, Government College University Faisalabad, Faisalabad, Pakistan

**Keywords:** hydrogel films, wound healing, antibacterial, Betula utilis, sodium alginate

## Abstract

Traditional wound dressings have a limited capacity to absorb exudates, are permeable to microbes, and may adhere to wounds, which leads to secondary injuries. Hydrogels are promising alternative dressings to overcome the above challenges. In this study, we developed sodium alginate-based hydrogel films loaded with Betula utilis bark extract. These films were prepared *via* solvent-casting crosslinking method and evaluated for wound healing activity. Prepared films were 0.05–0.083 mm thick, flexible with folding endurance ranging from 197–203 folds, which indicates good physical properties. Optimized formulations exhibited successful loading of extract in the film matrix without any interaction as confirmed by FTIR. Maximum zone of inhibition against Gram-positive and Gram-negative bacteria was achieved by optimum formulation (B6), i.e., 19 mm and 9 mm, respectively, with > 90% scavenging activity. Furthermore, this optimum formulation (B6) was able to achieve 93% wound contraction in rats. Histograms of the optimized formulation treated group also revealed complete reepithelization of wounds. Conclusively, our extract-loaded hydrogel dressing successfully demonstrated its potential for cutaneous wound healing.

## Introduction

The skin acts as a primary barrier against external pathogens with sensory, thermoregulatory, and protective functions. Thus, it plays a vital role in the wellbeing and integrity of living organisms. General skin injuries to organisms require immediate and effective treatment (Yang et al., 2019). Wound healing represents a series of events related to immunological and physiological phenomena, for instance, inflammation, propagation, remodeling, and scar formation. The healing process can be delayed due to diverse pathologies among different wounds, such as post-operative wounds, burns, chronic ulcers, etc. (Grambow et al., 2021). Furthermore, age, disease, infection, stress, and nutrition also contribute to a delayed healing process. Such cases are of great concern and should be treated with effective remedies (Guo and DiPietro, 2010) to reduce the suffering of patients and the overall cost of treatment.

Traditional wound healing biomaterials act as an active transient obstacle to stop bleeding and avoid infections. Any improvement in biomaterials properties could be helpful to accelerate the wound healing process. A wound dressing is considered ideal if it is able to maintain a moist environment, absorb unwanted exudates, and protect the wound site from infections (Zhang et al., 2018). The therapeutic worth of these wound dressings is subject to diverse factors, such as the type and ratio of polymers used, the behavior of the cross-linker, the nature of formulation additives, the methodology adopted, and the selection of suitable antibacterial agents (Aderibigbe and Buyana, 2018).

In comparison to traditional dressings, film-based dressings are flexible, transparent, and adhesive (Mahmood et al., 2021; Tenorová et al., 2022). Although they cannot absorb fluid, they are able to ‘breathe off’ small amounts of fluid by moisture vapor transpiration (Yazdanpanah et al., 2015). Currently, special attention is being paid to developing hydrogel-based films, owing to their intrinsic capacity to hold liquid content and provide a moist environment at the wound site for accelerated healing (Straccia et al., 2015). Hydrogels are polymeric systems having networks of hydrophilic polymers developed *via* chemical, ionic, physical, and hydrogen bonding. In contrast to synthetic polymers, hydrogels developed with natural polymers are more economical, biocompatible, and biodegradable and are easily modified for diverse applications (Mali et al., 2018).

Alginate is a biopolymer with numerous biomedical uses owing to its biocompatibility, non-toxic nature, and ease of availability. These attributes are also favorable for wound healing applications (Lee and Mooney, 2012). Alginate-based hydrogel dressings have the potential to soak up unnecessary wound fluid, maintain the body’s natural hydration status, and reduce bacterial load at the wound bed (Koehler et al., 2018). Cross-linking of alginate is primarily due to blocks of D-mannuronic (M) and L-guluronic (G) acids. The last mentioned block can be involved in gelation *via* complexion with divalent cations. These blocks vary depending on the position of the monomer in a chain, either homo polymeric (MM or GG) or hetero polymeric (MG or GM) (Ibrahim et al., 2019). Moreover, the physical properties of alginate gels are related to their composition (i.e., M/G ratio), sequence, G block length, and molecular weight, while the mechanical properties of alginate-based gels are strengthened by increasing the length of the G block and their molecular weight (Lee and Mooney, 2012; Liu et al., 2017). The literature shows that various groups have incorporated plant extract in sodium alginate films. Purple onion peel extract was loaded into sodium alginate films, which enhanced its thickness and antioxidant activity (Santos et al., 2021). In another study, green propolis extract and silica nanoparticles were loaded into sodium alginate-based films. The presence of propolis extract imparted a strong UV light-blocking effect and a DPPH radical scavenging activity (Marangoni Júnior et al., 2022). Guava leaf extract possesses antioxidant and antibacterial activities and authors showed its incorporation into sodium alginate films enhanced tensile strength, and antioxidant and antimicrobial activity of the films (Luo et al., 2019). Apart from the above studies, authors have incorporated various plant extracts in alginate films, such as *Hypericum perforatum* extract for wound healing (Mutlu et al., 2022) and roselle extract with good antibacterial activity with more pronounced effects on Gram-positive bacteria with increasing concentrations of the extract (Aydin and Zorlu, 2022).

Betula utilis is widely distributed in the Himalayas. The bark of the plant contains triterpenoids, such as betulin, betulinic acid, and lupeol, which are reported to have potent anticancer, antioxidant, anti-inflammatory, and antimicrobial properties against various pathogenic bacterial species (Verma et al., 2014). Triterpenoids, such as betulin, deal with the inflammatory phase by regulating several pro-inflammatory mediators, resulting in enhanced migration of keratinocytes, which is essential in the second phase of wound healing. It has been observed that triterpene extract of birch bark showed good results in scratch assay tests using primary human keratinocytes. In these, authors reported upregulation of various pro-inflammatory cytokines, chemokines, and cyclooxygenase 2 on gene and protein levels (Ebeling et al., 2014). In another study, birch bark extract was loaded into a lecithin-based nanoemulsion that showed good results in scratch assays (Vater et al., 2022). Oleogel-S10 loaded with 10% triterpene dry extract from the bark of *Betulae* cortex showed faster re-epithelialization of wounds in patients with dystrophic epidermolysis bullosa (Schwieger-Briel et al., 2017). Therefore, in this study, we aimed to develop alginate-based hydrogel films containing Betula utilis bark extract and assess its wound healing efficacy.

## Materials and methods

### Materials

The bark of Betula utilis was procured from Pansar Inn herb store, Karachi, Pakistan (Identified by the Department of Botany, Government College University Faisalabad, Pakistan *via* voucher No 298-bot-21). Sodium alginate (CAS No 9005–38–3) was purchased from DAEJUNG chemicals Co. LTD., Korea. Propylene glycol (PG) (CAS No 57–55–6), calcium chloride (CAS No10043-52–4), and tween 80 (CAS No 9005–65–6) were procured from Sigma Aldrich, United States of America. All other chemicals used in this study were of analytical grade and utilized as such.

### Extraction of plant extract

The plant bark was cut into small pieces and ground. The powdered bark (18 g) was extracted with 95% ethanol (500 ml) at 40°C for 72 h in a soxhlet extractor and later concentrated by using a rotary evaporator. The yellowish-brown residue was air-dried for 4–5 days and used for subsequent experiments (Joshi et al., 2013).

### Preparation of hydrogel films

The films were developed by a slight modification of the previously reported solvent casting technique (Pereira et al., 2013). Initially, a 2% w/v solution of sodium alginate was prepared in distilled water by continuous stirring for half an hour at 700 rpm at 50°C. Then, propylene glycol (15% w/w mass of polymer) was added to the above polymeric solution. Later, different concentrations of bark extract in ethanol were slowly added to the polymeric solution (Table 1) with continuous stirring for 20 min at 700 rpm.

**TABLE 1:** Composition of sodium alginate hydrogel films.

Code	B. Utilis (mg)	Ethanol (mL)	Sodium alginate (2% w/v)	CaCl2 (0.5% w/v)
B0 (Blank)	-	-	200mg	50mg
B1	50	1	200mg	50mg
B2	100	1	200mg	50mg
B3	150	1	200mg	50mg
B4	200	2	200mg	50mg
B5	300	2	200mg	50mg
B6	400	2	200mg	50mg

^a^Each formulation contains propylene glycol (15% w/w of sodium alginate) and tween 80 (0.2% w/v).

The above solution was cast into a glass petri dish and calcium chloride (0.5% w/v) solution was sprayed from a distance of 6 cm. The films were allowed to dry at room temperature. Finally, the dried films were peeled out, wrapped in aluminum foil, and stored in desiccators until further testing.

### Hydrogel film characterization

#### Film thickness, folding endurance, and weight variation

The thickness of hydrogel films was evaluated by using a digital micrometer (Shang Chong Co. Ltd., China). The thickness of each film was noted at ten random positions on each film (Pereira et al., 2013). The folding endurance of each film was tested by repeatedly folding the film at the same place until they broke (Saranya and Manoj, 2016). In weight variation, ten random samples (2 × 2 cm^2^) from each film formulation were weighed (OHAUS adventurer analytical balance, United States of America). All results were reported as mean plus minus standard deviation (SD).

#### Swelling studies

The swelling behavior of films was determined by the gravimetric technique. Initially, the weight of the dried hydrogel films (2 × 2 cm^2^) (W_D_) was noted. Then, each sample was immersed in a petri dish containing phosphate-buffered saline (PBS) (15 ml) of pH 7.4. At different time intervals, the swollen films were withdrawn from the PBS and the weight of the swollen films (W_S_) was noted until equilibrium after removing excess surface water. The swelling efficiency (%) was calculated by using the following equation:

$$Swelling\;efficiency\left(\%\right)=\left[\frac{W_{S}-W_{D}}{W_{S}}\right]\times 100$$

W_S_ = weight of the swollen gel, W_D_ = weight of a dried gel (Huber et al., 2018).

Swelling data for each film was collected in triplicate.

#### Fourier transform infrared spectroscopy (FTIR)

A FTIR (Model: FTIR-7600, Guangdong, China) analysis of pure extract and hydrogel samples was performed in the range of 4000 to 500 cm^−1^ to investigate any possible interaction among formulation ingredients and extract (Wu et al., 2017).

#### X-ray diffraction (XRD)

X-ray diffraction patterns of extract and hydrogels were obtained by scanning them on an X-ray diffractometer (Model: D8 advance, Bruker, US) at 2θ from 5–60° (Mishra et al., 2011). These patterns were analyzed to assess the amorphous or crystalline nature of the given sample.

#### Scanning electron microscopy (SEM)

The extract and prepared hydrogel films were observed under an electron microscope (Model: Cube series company Emcrafts, Korea) to assess their surface morphology (Sun et al., 2017).

#### Antibacterial activity

The hydrogel films were assessed for antimicrobial activity against *Staphylococcus aureus* (Gram-positive) and *Escherichia coli* (Gram-negative) by using the disc diffusion method. Standardized inoculum of respective bacteria were activated in tryptic soy agar (TSA) for 24 h and incubated at 37°C until the count reached 1 × 10^9^ CFU per mL. Afterward, 0.5 ml of the standardized inoculum was dispersed on the surface of the nutrient agar plates. Then, films of specified dimensions of10 mm were placed on the plates. Each Petri plate was incubated for 24 h at 37°C and, later, the zone of inhibition was noted (El-Kased et al., 2017).

#### Antioxidant assay

For the DPPH analysis, prepared solutions of films (5 mm) and pure extract (0.4 g) in ethanol were individually added to 2.5 ml of 0.1 mM DPPH in ethanol. Ethanolic DPPH solution was used as blank. Afterward, they were incubated in darkness for 30 min. Finally, the absorbance of the mixture was noted at 517 nm on a UV–vis spectrophotometer in triplicate (Cecil instrument, Cambridge England) (Liu et al., 2016). Results were calculated as follows:

$$\%\;scavenging\;activity=\frac{{Abs}_{blank}-{Abs}_{sample}}{{Abs}_{blank}}\times 1OO$$

### *In vivo* wound healing assessment

Wound healing was studied on the excision wound model in albino rats. The Ethical Review Committee (ERC) of the Government College University Faisalabad, Faisalabad, Pakistan, approved all the protocols (Ref. No: GCUF/ERC/42). All rats were acclimatized for 1 week in an animal house at a relative humidity of 55 ± 5%, temperature of 25°C ± 2°C, in a 12 h of light/dark cycle, and given proper feed with water *ad libitum*. All rats were divided into four groups (n = 4) and kept in separate cages with different color tags for proper identification. Rats were anesthetized by intraperitoneal injection of ketamine (0.05 mL/g) and xylocaine (0.01 mL/g) and their skin on the dorsal side was shaved. Afterward, the skin was cleaned with a spirit swab and a 5 mm wound was created with the help of a sterile biopsy punch. After that, a section of prepared hydrogel film was placed on each wound and secured with an elastic adhesive bandage. The size of each wound was noted with a digital Vernier caliper and pictures were also taken on days 0, 2, 4, 6, 8, and 10. The closure rate of each wound was estimated by the following equation:

$$Wound\;closure\;\left(\%\right)=\left[\frac{A_{n}}{A_{0}}\right]\times 100$$

Here, “A_0_” is the original wound size at day zero; “A_n_” wound area size at days 0, 2, 4, 6, 8, and 10 (Zhang et al., 2018).

### Histopathological examination

Skin from the wound bed along with adjacent normal skin of euthanized rats was fixed in 10% buffered formalin, embedded in paraffin, and sectioned (4–6 µm) by using a microtome. Afterward, tissue sections were stained with hematoxylin-eosin (H&E) to evaluate epidermal regeneration. Prepared slides were observed under a trinocular microscope fitted with a 5-megapixel camera (Accuscope3000, United States of America) (Beyranvand et al., 2019).

### Statistical evaluation

To compare results, statistical evaluation was carried out by one-way ANOVA with a *post hoc* Tukey test with a “*p*” value of less than 0.05 on Graphpad prism 5, and results are expressed as the mean ± S.D. for each analysis.

## Results and discussion

### Thickness, folding endurance, and weight variation studies

The thickness of all prepared films was analyzed and their results are shown in Table 2. The thickness of all films lay in the range of 0.054 ± 0.001 mm to 0.083 ± 0.003 mm and their weights ranged from 0.17 ± 0.002 g to 0.79 ± 0.003 g. Results showed that as the extract concentration increased in films, the thickness and weight of the films also increased, which was in agreement with previously published results (Junmahasathien et al., 2018). These parameters also suggest the suitability of the method to prepare extract-loaded films with uniform content and thickness, which is essential for mass production. Folding endurance represents the capability of the film to resist breakage, i.e., the higher the folding endurance, the less breakage will happen. The folding endurance of tested films was 197 ± 8 to 203 ± 5 folds, indicating that these films possess good mechanical properties (Saranya and Manoj, 2016) to withstand transportation stresses and will not break when applied to wounds. Moreover, the presence of the extract did not affect the folding endurance.

**TABLE 2:** Thickness, weight variation, and folding endurance of hydrogel films (n = 10).

Code	Thickness (mm) ± SD	Weight variation (g) ± SD	Folding endurance
B0	0.054 ± 0.001	0.17 ± 0.002	203 ± 7
B1	0.058 ± 0.003	0.25 ± 0.003	199 ± 5
B2	0.062 ± 0.005	0.39 ± 0.003	196 ± 7
B3	0.068 ± 0.002	0.41 ± 0.001	197 ± 5
B4	0.071 ± 0.002	0.52 ± 0.004	201 ± 8
B5	0.079 ± 0.006	0.63 ± 0.001	205 ± 7
B6	0.083 ± 0.003	0.79 ± 0.003	199 ± 5

### Swelling behavior of hydrogel films

**FIGURE 1:**
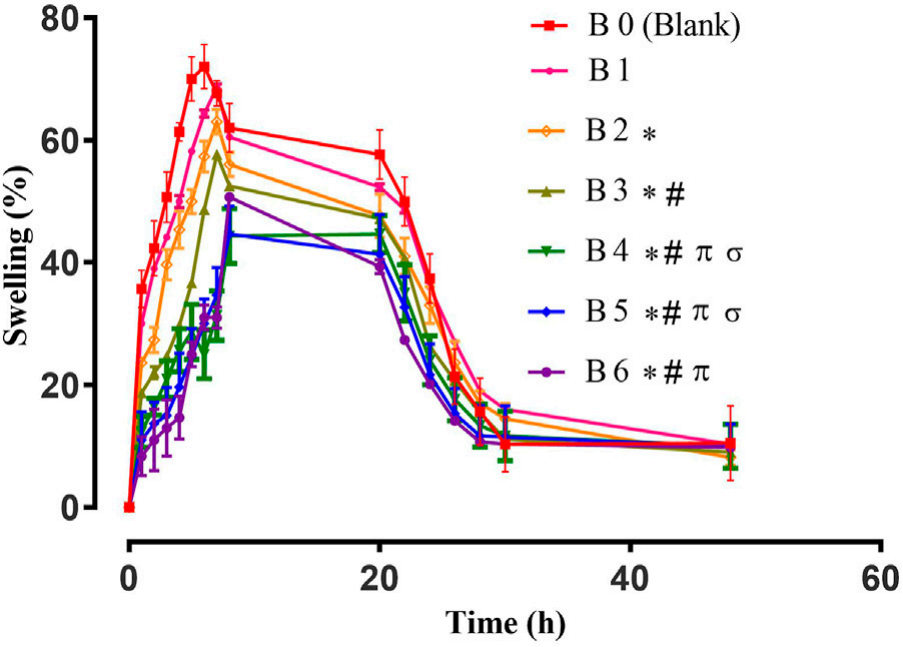
Swelling study of Hydrogel films. Error bar indicates the standard deviation (n = 3, *p*-value <0.05 for the highest swelling achieved by the formulations, * compared with B0 (Blank), # compared with B1, π compared with B2, σ compared with B3).

The swelling behavior of hydrogel films is very important as it affects the release rate of the encapsulated extract and its activity. A slow swelling ratio prolongs the release and *vice versa*. Therefore, swelling behavior is a vital parameter to determine the end application of prepared hydrogel films (Wang et al., 2020). The swelling patterns of blank and extract-loaded hydrogel films are elaborated in Figure 1.

Different parameters can affect the swelling behavior of hydrogel films. One of them is the concentration of the cross-linker (Bajpai et al., 2015). On the other hand, the hydrophilic/hydrophobic nature of polymers and loaded moieties also alter the swelling pattern of hydrogels. All tested films displayed maximum swelling for up to 8 h and afterward their swelling decreased, which might be due to polymeric erosion as observed in previous studies (Sharma et al., 2016; Mahmood et al., 2021). For blank hydrogel films, higher percent swelling was observed due to enhanced hydrophilicity of the sodium alginate network (Jayaramudu et al., 2013). The swelling of extract-loaded films was reduced, which might be due to the presence of hydrophobic constituents of plant extract (Wang et al., 2020). For wound healing, the swelling of hydrogel plays an important role as it not only controls the release of entrapped active constituents but also helps in the adhesion of the hydrogel film to the wound, the extent of exudates absorbance, and the maintenance of optimum moisture content at wound bed, which stimulates fibroblast proliferation and keratinocyte migration. Ultimately, these factors are crucial for the complete epithelialization of the wound (Mahmood et al., 2021).

### Antibacterial studies

Antibacterial activities for all formulations were assessed against *Staphylococcus aureus* and *Escherichia coli* by measuring the respective zone of inhibition. All results are listed in Table 3.

**TABLE 3:** Antibacterial activity of Betula utilis extract-loaded hydrogel films (n = 3).

Code	Betula utilis extract (mg)	Zone of inhibition (mm ± SD) (S. aureus)	Zone of inhibition (mm ± SD) (E. coli)
B0	0	-	-
B1	50	-	-
B2	100	-	-
B3	150	-	-
B4	200	9 ± 0.002	-
B5	300	17 ± 0.005	-
B6	400	19 ± 0.003	9 ± 0.006

**FIGURE 2:**
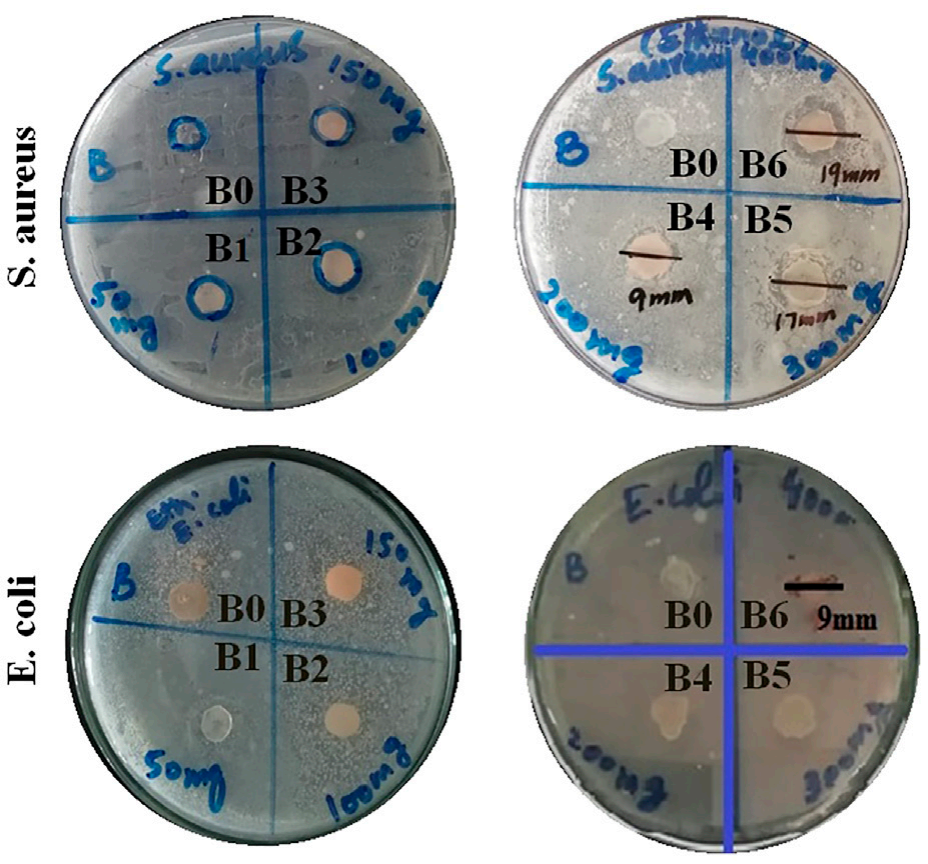
Antibacterial activity at different extract concentration-loaded hydrogel films against *S. aureus* and *E. coli* bacteria.

The antibacterial activity of hydrogel film was improved by loading plant extract. Moreover, antibacterial effects were enhanced by increasing the concentrations of the plant extract (Debalke et al., 2018). From the above data, it became evident that formulations B4, B5, and B6 showed concentration-dependent antibacterial activity. Among them, maximum bacterial inhibition was achieved by B6 with a zone of inhibition of 19 mm and 9 mm against *S. aureus* and *E. coli,* respectively, as shown in Table 3 and Figure 2. Moreover, the formulation containing plant extract was more effective against *S. aureus* (Gram-positive) when compared with *E. coli* (Gram-negative). This was primarily due to dissimilarity in their cell wall structure as Gram-negative bacterial cell walls have an additional hydrophilic lipo-polysaccharide layer that hinders the penetration of hydrophobic components (Adeli et al., 2019; Mahmood et al., 2021).

Studies revealed the presence of triterpene, such as betulin, betulinic acid, oleanolic acid, and lupeol, in Betula bark, whichshowed significant antibacterial activity against different bacterial species, especially *S. aureus* and *E. coli* (Duric et al., 2013). In one study, the researcher tested plant extracts of Syzygium aromaticum, Cuminum cyminum, Punica granatum, Zingiber officinales, and Thymus vulgaris against different bacterial strains and found concentration-dependent antimicrobial activity (Mostafa et al., 2018). Polyvinyl alcohol hydrogel was loaded with guava leaf extract and it was reported that a higher zone of inhibition against *S. aureus* and *P. aeruginosa occurred with increasing concentrations of plant extract (* *Waresindo et al., 2021* *). In another study,* carbomer-based hydrogel wound dressings were developed by using ethanolic extracts of *Rosmarinus officinalis* aerial parts, *Achillea millefolium,* and *Calendula officinalis* flowers. The researchers reported better antibacterial activity for *Rosmarinus officinalis* extract and the one containing the blend of all extracts; thus, showing potential for future wound healing dressing (Gavan et al., 2022).

After carefully evaluating the preformulation data, formulation B6 was considered the optimum formulation due to considerable antibacterial action, folding endurance, and optimum swelling. However, for further testing, B4 will be analyzed along with B6 for comparison as it contains half the quantity of plant bark extract. Furthermore, during wound healing studies it helps to establish a dose-response relationship with increasing concentration of extract.

## FTIR

**FIGURE 3:**
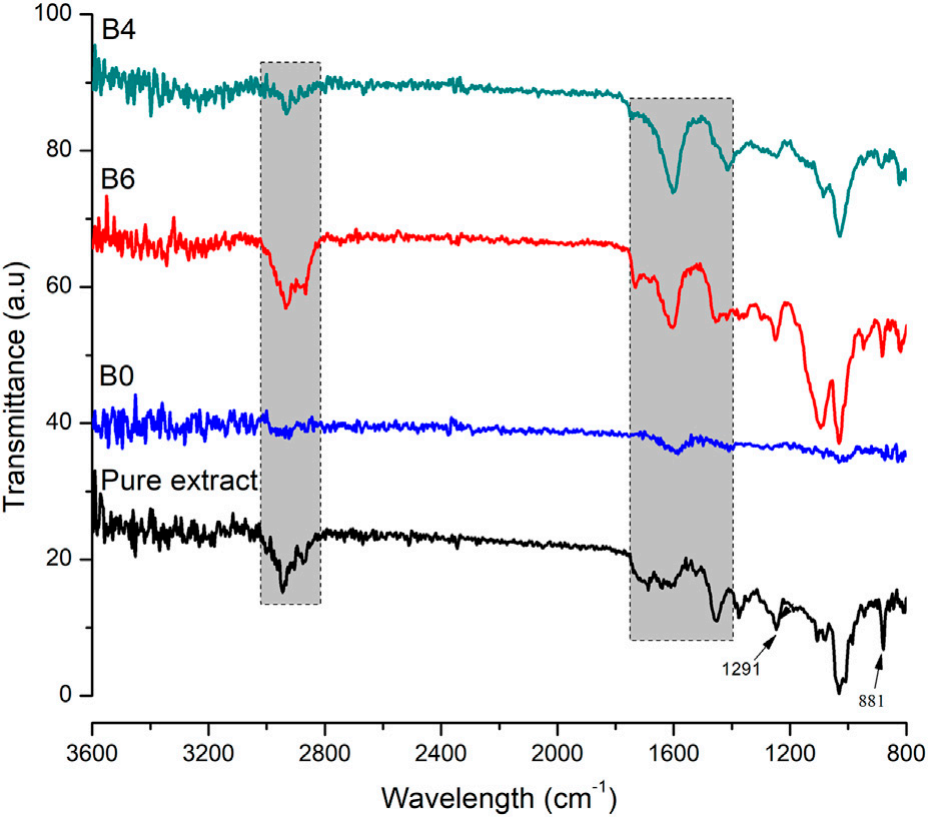
FTIR spectra of pure extract, blank (B0), B6, and B4. The shaded region shows important peaks of extract.

To find any possible interaction among polymer and loaded components of hydrogel films, FTIR analysis was performed on pure extract, blank, and extract-loaded hydrogel films as shown in Figure 3. Sodium alginate in blank film displayed stretching vibrations of O–H in the range of 3000–3600 cm^−1^. Stretching of the aliphatic C–H group was found between 2920 and 2850 cm^−1^. Bands found at 1649 and 1460 cm^−1^ were accredited to stretching vibrations of carboxylate ions. The bands at 1107 and 935 cm^−1^ were observed due to C–O vibration stretching of the pyranosyl ring and the C–O stretching of sodium alginate (Daemi and Barikani, 2012). FTIR spectrum of the pure extract showed characteristic peaks of the main constituent, i.e., betulin at 3430, 2968, 1716, 1641, 1600, 1581, 1291, and 881cm^−1^ (Cîntă-Pînzaru et al., 2012). Distinctive peaks of extract and sodium alginate were observed in the developed hydrogel films. Therefore, it can be summarized that extract-loaded films were developed without any interaction.

### XRD analysis

**FIGURE 4:**
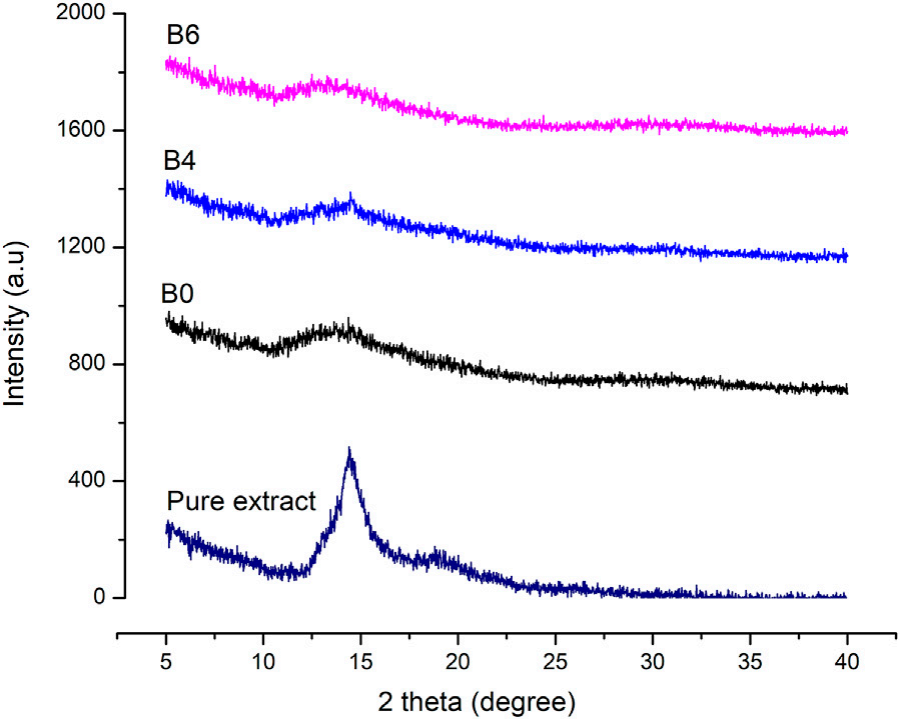
XRD graphs of pure Betula utilis extract and optimized formulations loaded with plant extract.

X-ray diffraction was performed to assess the physical state of samples (either amorphous or crystalline). The diffractograms of the pure extract were compared with prepared films and are represented in Figure 4.

Characteristic peaks of Betula utilis extract in the XRD graph indicate the crystalline form of the active constituent as reported previously for Betula pendula (Soto-Salcido et al., 2020). Moreover, blank as well as extract-loaded formulations (B4 and B6) showed amorphous behavior when compared to pure extract. The extract loading in alginate hydrogel film might result in higher surface disorders that increase their solubility in comparison to the crystalline materials (Cheng et al., 2014).

## SEM

**FIGURE 5:**
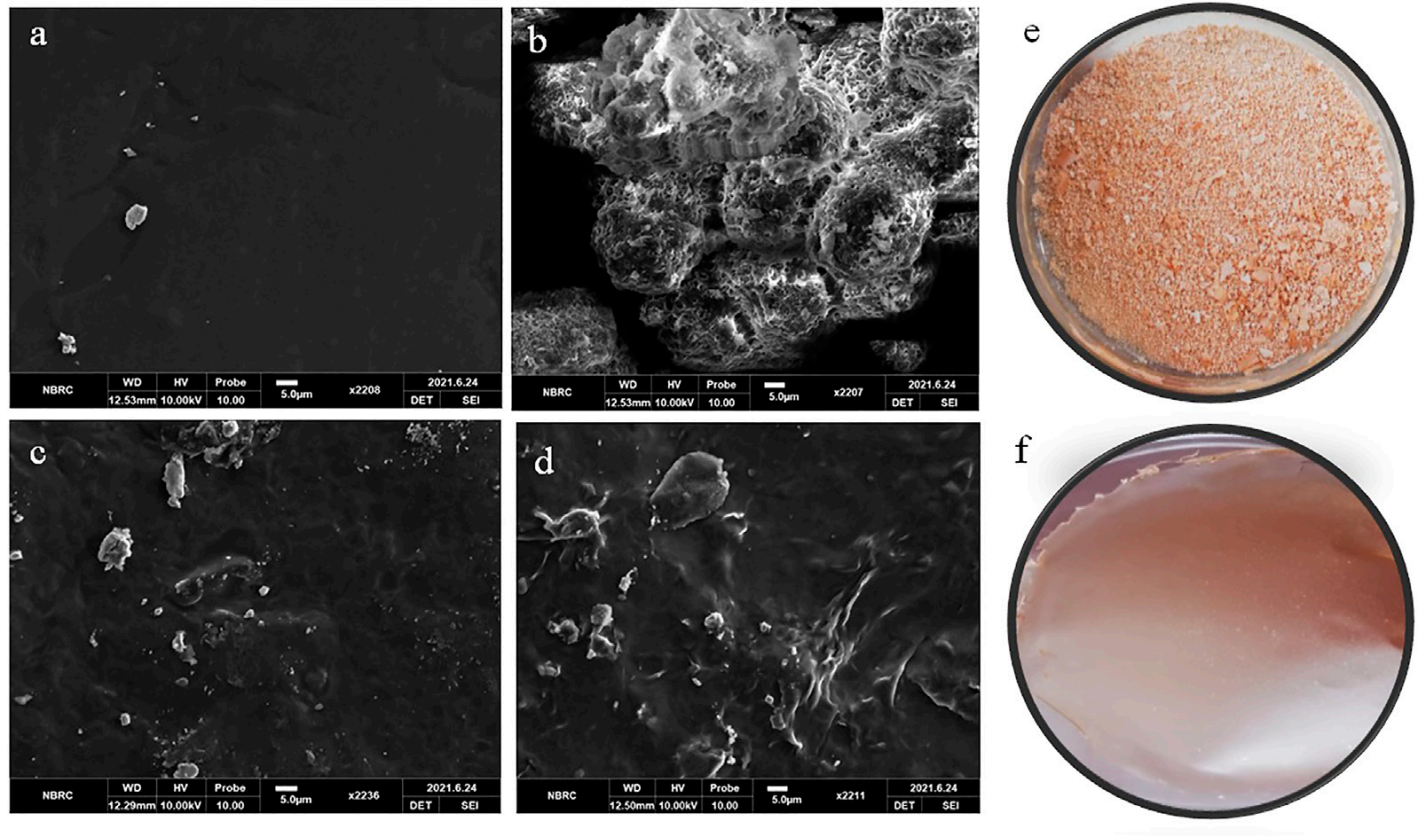
SEM images of **(A)** blank film (B0), **(B)** pure Betula utilis extract, **(C)** B4, and **(D)** B6. Optical image **(E)** pure Betula utilis extract and **(F)** B6.

Surface characteristics of pure extract, blank, and extract-loaded films were determined by taking microscopic images, as shown in Figure 5.

The blank film surface was smooth and non-porous, with few defects and wrinkles (Figure 5A). These defects might be due to cross-linking of polymeric chains by calcium chloride or aggregation and collapse of the polymeric network during drying. Similar observations were reported for gellan gum and sodium alginate hydrogel films (Badita et al., 2020; Mahmood et al., 2021). The optical image of pure extract shows it is a powder with aggregates (Figure 5E). SEM of pure extract (Figure 5B) revealed that particles were aggregated in spherical form. For extract-loaded hydrogel films (B4 and B6), the microscopic analysis revealed rough, irregular surfaces with particles, which increased with increasing concentration of extract. This roughness was attributed to the uniform distribution of extract that ensures therapeutic benefits. The presence of surface particles can be explained by the following facts. Upon loading, extract molecules are loaded in the hydrogel film network. However, as the quantity of extract is increased, the excess quantity may deposit on the surface of hydrogel films, especially during drying. Similar observations were reported for glutaraldehyde crosslinked poly (vinyl alcohol)/poly (vinylpyrrolidone) films where *H. sabdriffol*, *C. pepo*, *T. indica,* and *L. nobilis* extracts were loaded (Soud et al., 2020).

### Antioxidant assay

**FIGURE 6:**
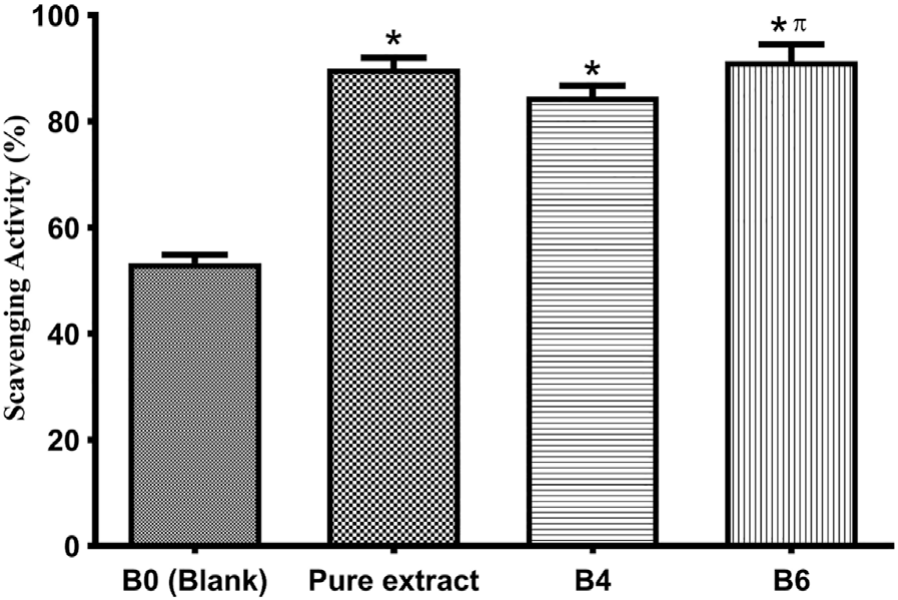
Antioxidant activity of the pure extract, optimized formulations (B4 and B6), and blank (B0). Error bar represents the standard deviation (n = 3; *p*-value ˂0.05, *compared with Blank, and π compared with B4).

DPHH assay was developed by Blois (Blois, 1958) to assess the antioxidant activity by employing a stable free radical, i.e., α, α-diphenyl-β-picrylhydrazyl (DPPH; C_18_H_12_N_5_O_6_, M = 394.33). This assay measures the scavenging capacity of antioxidants towards it. Antioxidants are widely found in biological systems and naturally in different herbs, plants, oils, etc. A balance between oxidants and antioxidants is very important for human health (Kedare and Singh, 2011). In this study, we estimated the scavenging activity of pure extract and optimized hydrogel films by DPPH analysis (Figure 6). It is observed that antioxidants are responsible for controlling wound oxidative stress. A high level of Reactive Oxygen Species (ROS) increases oxidative stress by damaging cells while a low level of ROS, and it not only protects the tissues against infection but also stimulates wound healing by the production of cell surviving signaling (Comino-Sanz and López-Franco, 2021). The pure extract exhibited free radical scavenging ability. Moreover, extract-loaded films have also shown comparative antioxidant activity, which indicates extracts have maintained their activity in hydrogel films. For loaded films, the antioxidant activity slightly increased with increasing concentration of extract. It is believed that flavonoids, mainly quercetin present in plants, have free radical scavenging potential (Pandey et al., 2016; Comino-Sanz and López-Franco, 2021). A group of researchers tested Betula pendula leaf extract for antioxidant activity. They observed that this activity was time and concentration-dependent. With 10 μg/ml extract, antioxidant activity was 41.03% after 30 min. As the concentration of the extract was increased the antioxidant activity also increased. For instance, 200 μg/ml of leaf extract gave 98.47% activity after 15 min, which raised to 99.46% when the reaction time was 30 min (Penkov et al., 2018).

### *In vivo* healing effect of hydrogel films

**FIGURE 7:**
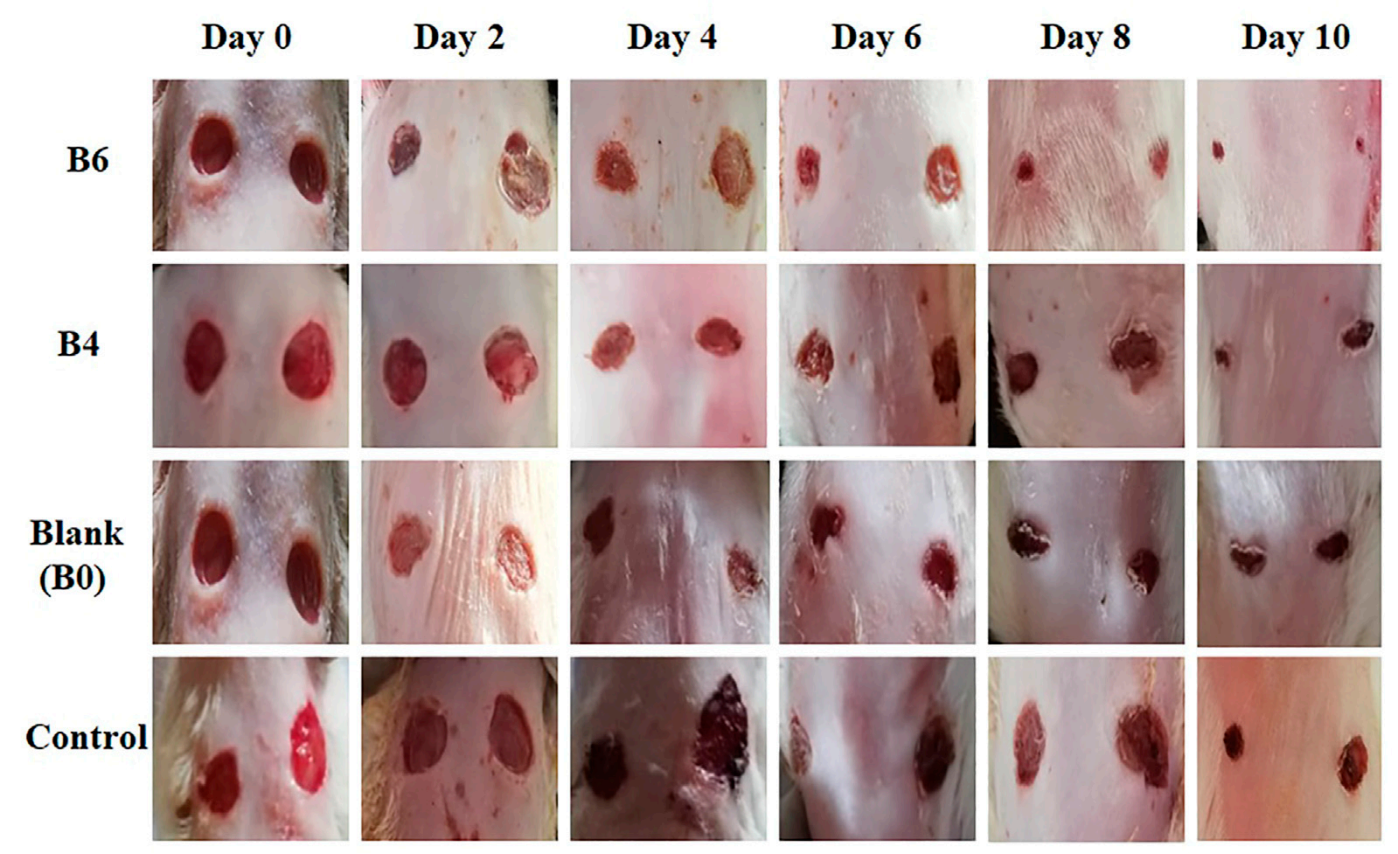
Macroscopic view of wound size reduction in various groups on different days i.e., group treated with extract-loaded films (B6 and B4), blank film (B0), and control (n = 4).

**FIGURE 8:**
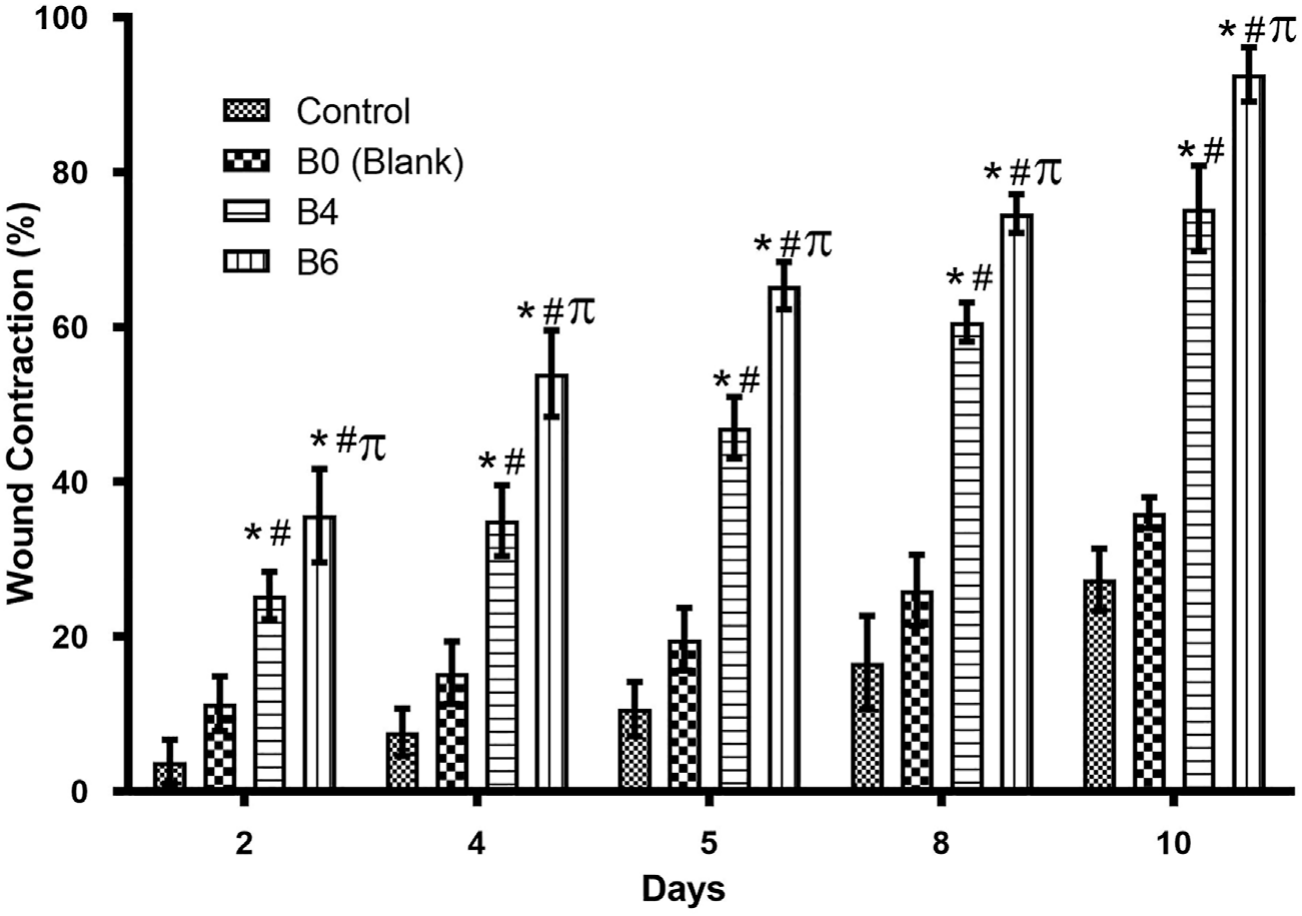
Comparison of wound healing activity of different formulations on different days. Error bar represents the standard deviation (n = 4; *p*-value ˂0.05, *compared with control, # compared with Blank, and π compared with B4).

The healing efficacy of prepared formulations was assessed on rats with full-thickened wounds. The percentage of wound contraction was calculated for rats treated with hydrogel films loaded with extract (B4 and B6) and without extract, i.e., blank (B0). Later, results were compared with the control group, as shown in Figure 7 and Figure 8.

Quantitative analysis (Table 4) of wounds showed significant wound contractions in groups treated with B4 and B6 as compared to the control and blank film-treated groups. On the fourth day, B6 and B4 showed 54 ± 0.2 and 35 ± 0.2 percent wound contraction, respectively. In comparison, the blank and control groups showed 15 ± 0.1 and 8 ± 0.4 percent wound contraction on the fourth day. On the 10th day, the B6 treated group showed almost 100 percent wound contraction, while the B4 group demonstrated 74 percent wound contraction. In the blank film-treated and control groups, wound contraction was less than 50 percent, although the blank group showed more contraction than the control group. This was attributed to multiple reasons as hydrogels are three-dimensional structures that provide optimum moisture, mimic the skin, act as a barrier for invading microorganism, and, more importantly, can be easily removed from wounds without damaging underlying cells (Khan et al., 2021). Furthermore, sodium alginate also contributes to hemostasis and wound healing due to good biocompatibility, mucoadhesion, high swelling, and oxygen permeability (Aderibigbe and Buyana, 2018; Xie et al., 2022). The literature survey revealed that phytochemical constituents, such as flavonoids and triterpenoids, are known to promote the wound healing process owing to astringent and antimicrobial properties. It was reported that 97% of the birch Betula extract is composed of pentacyclic triterpenes. Among all, 87% of betulin is present as a key component with wound healing potential (Ebeling et al., 2014). In the case of our films, increasing the concentration of extract also enhanced wound contraction. Our results are supported by previous studies where Melia azedarach extract loaded poly (vinyl alcohol)/pectin hydrogel was developed for the treatment of burn wounds. During an *in vitro* scratch test, treatment with 50 μg/ml of Melia azedarach showed good recovery of wounds as compared to 10 μg/ml of Melia azedarach (Lee et al., 2022). The literature revealed that betulin-enriched extract from birch bark was loaded in nanoemulsion. This formulation was able to close the gaps in the scratch assay test using fibroblasts and keratinocytes. Betulin can promote wound healing by influencing three stages of the healing process. First, it temporarily stimulates inflammatory mediators, for instance, cyclooxygenase-2, IL-6, or IL-8, which attracts macrophages, phagocytes, and granulocytes to initiate wound bed cleaning. During the second stage, betulin promotes skin cell migration by inducing IL-6 and signal transducer and activator of transcription 3 (STAT3). They affect the outermost cells of the skin, i.e., keratinocytes, by increasing the formation of lamellipodia, filopodia, and stress fibers. Such structures are developed when cells are stimulated to migrate until they come into contact with each other and accelerate re-epithelialization. In the last stage, betulin stimulates various differentiation markers, such as involucrin, keratin 10, and transglutaminase, which differentiates cells and helps in the maturation and remodeling of wounds (Vater et al., 2022).

**TABLE 4:** % wound contraction of various formulations on different days (n = 4).

Code	Percent wound contraction					
	Day 0	Day 2nd	Day 4th	Day 6th	Day 8th	Day 10th
B6	0	36 ± 0.1	54 ± 0.2	65 ± 0.4	75 ± 0.1	93 ± 0.5
B4	0	25 ± 0.5	35 ± 0.2	47 ± 0.8	61 ± 0.2	74 ± 0.6
B0 (Blank)	0	11 ± 0.2	15 ± 0.1	20 ± 0.4	26 ± 0.7	36 ± 0.2
Control	0	4 ± 0.1	8 ± 0.4	11 ± 0.1	17 ± 0.3	27 ± 0.9

### Histology

**FIGURE 9:**
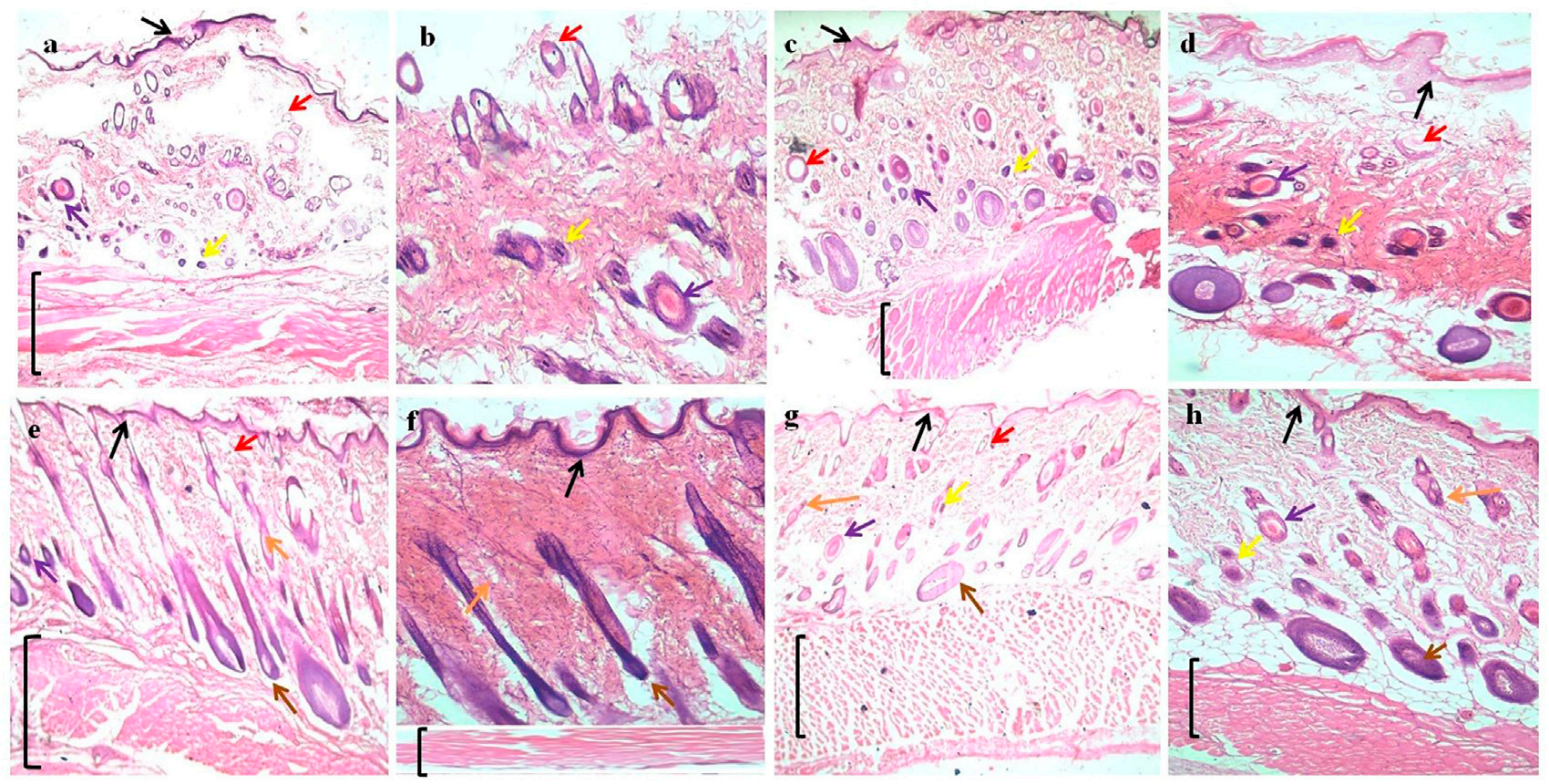
H&E (hematoxylin and eosin) straining of wound tissues micrographs; **(A)** and **(B)** control at 4x and 10x, **(C)** and **(D)** blank (B0) at 4x and 10x, **(E)** and **(F)** optimized formulation (B4) at 4x and10x, and **(G)** and **(H)** optimized formulation (B6) at 4x and 10x. Arrows indicate: Black = epidermis, Red = blood vessel, Purple = macrophages, Yellow = inflammatory cells, Orange = sebaceous gland, Brown = hair follicles, and Black bracket = connective tissue.

To observe histological changes, sections of skin tissue were taken and stained by H&E. The groups treated with optimized formulation exhibited excellent healing of wounds with the visible recovery of the epidermis as compared to the blank and control groups, as shown in Figure 9.

The absence of superficial epithelium, few hair follicles, clusters of degenerated neutrophils, necrotic alterations, and the perseverance of inflammatory exudates were also observed in both control and blank film groups, which indicates incomplete healing. For optimized formulation, better tissue formation, hair follicles, thick epidermis, and a significantly small number of inflammatory cells indicate better wound healing (Perini et al., 2015).

## Conclusion

Sodium alginate-based hydrogel dressings were successfully developed with Betula utilis bark extract. Hydrogel films loaded with plant extract exhibited optimum folding endurance, thickness, and antioxidant and antibacterial activity. The compatibility of plant extract with optimized formulations was verified by FTIR, and the amorphous nature of films was confirmed by XRD analysis. SEM analysis revealed a smooth surface of the blank film, but they became slightly rough after loading of extract. The swelling behavior of films was also assessed and it was found that with increasing concentration of extract swelling slightly decreased, which might be due to hydrophobic constituents of plant extract. Optimized formulations were tested in rats where extract-loaded films helped reduce wound healing time. The histopathological evaluation also confirmed this observation, with animal groups treated with optimized formulations (B4 and B6) having better tissue formation and reepithelization, which lead to better wound closure. As designed hydrogel dressings have successfully passed various *in vitro* and *in vivo* evaluations, they have, thus, been deemed fit for cutaneous wound healing. In the future, these dressings could be tested in more specific animal wound models for specific use.

## Data Availability

The original contributions presented in the study are included in the article, further inquiries can be directed to the corresponding authors.
